# Effect of warm intravenous and irrigating fluids on body temperature during transurethral resection of the prostate gland

**DOI:** 10.1186/1471-2490-7-15

**Published:** 2007-09-18

**Authors:** LI Okeke

**Affiliations:** 1 Urology Division, Department of Surgery, College of Medicine, University of Ibadan, University college Hospital, PMB 5116, Ibadan, Nigeria

## Abstract

**Background:**

Transurethral resection of the prostate gland with irrigation fluid at room temperature leads to perioperative hypothermia which could give rise to adverse cardiovascular events in the perioperative period. The use of isothermic irrigation fluid reduces but does not eliminate this risk. Routine use of warm intravenous fluids along with isothermic irrigation had not been documented. This study set out to investigate the effect of the use of warm intravenous fluid together with isothermic irrigation fluid on the body temperature in patients undergoing transurethral resection of the prostate gland.

**Methods:**

One hundred and twenty consented patients with obstructing benign prostatic hyperplasia were randomly assigned to one of 3 groups. Group 1 received irrigation and intravenous fluids at room temperature, group 2 received warmed irrigation fluid at 38°C along with intravenous fluid at room temperature while group 3 patients received warmed irrigation fluid and warmed intravenous fluids at 38°C. Their perioperative body temperature changes were monitored, analyzed and compared.

**Results:**

The mean decrease in body temperature at the end of the procedure was significantly greater in group 1 (0.98 ± 0.56°C) than in group 2 (0.42 ± .21°C) (p < 0.001). Significantly more patients in group 1 also experienced shivering. However, in group 3, there was no significant change in the mean body temperature (p > 0.05) and none of them felt cold or shivered.

**Conclusion:**

It is concluded that the use of isothermic irrigation fluid together with warm intravenous fluids during TURP prevents the occurrence of perioperative hypothermia.

**Trial registration number:**

CCT-NAPN-15944

## Background

Perioperative hypothermia is an unintentional drop in the core body temperature to less than 36°C during or immediately following a surgical operation [1]. Contributing factors vary and include extremes of age, low ambient room temperature, length and type of surgical procedure, use of cold irrigants and the type of anesthesia [1,2]. Perioperative hypothermia can have a wide range of underappreciated, detrimental effects. It increases left ventricular afterload, indicating increased myocardial work and oxygen demand which could result in myocardial ischaemia [3,4]. It induces shivering which has been shown to increase the oxygen consumption by as much as 500% [5]. This could be associated with post operative instability and prolonged recovery [3,6]. In patients with compromised cardiac function, there is increased risk of cardiac arrhythmias, angina pectoris and myocardial infarction in the subsequent 24 hours after surgery [7,8]. Studies by Jaffe et al [9] suggested that irrigation fluid temperature was not a factor responsible for altering the core temperature in patients undergoing transurethral resection of the prostate (TURP). However, many other studies before this had conclusively shown that continuously warmed irrigating solutions could prevent the fall in body temperature which occurs during TURP [2,10,11].

In 1995 when TURP under caudal block regional anesthesia with boiled water as irrigation fluid [12], was commenced at our centre, some of the patients experienced shivering. This usually occurred towards the end of their procedure or in the immediate postoperative period, requiring external warming with warm blankets. When we commenced using warm irrigating fluids and intravenous fluids at room temperature, some of the patients still shivered but when we used warm irrigation fluids and warm intravenous fluids, shivering was abolished. We therefore decided to objectively document these observations by designing this prospective randomized study in order to subject our observations to statistical analysis.

## Methods

Between January 2001 and December 2005, a total of 120 patients scheduled for transurethral resection of the prostate gland at our institution were enrolled into this randomized study. The study was approved by the hospital ethical review committee and all 120 patients provided written informed consent. The patients were randomly assigned to one of 3 groups by picking a ballot. Group 1 received irrigation and intravenous fluids at room temperature, group 2 received warmed irrigation fluid at 38°C along with intravenous fluid at room temperature while group 3 patients received warmed irrigation fluid and warmed intravenous fluids at 38°C. Previously boiled water which was allowed to cool was the irrigation fluid used for all groups. For groups 2 and 3, the appropriate temperature was achieved by mixing previously boiled and cooled water with varying amounts of freshly boiled water. The intravenous fluid for group 3 was warmed by immersing 4 bags of 500 ml intravenous fluids in a bucket containing warm water at 45°C. Only normal saline was used as intravenous fluid. Patients with co-morbid conditions such as diabetes mellitus, inguinal hernia, vesical calculi, asthma, axial skeletal or hip deformities which interfered with Lloyd Davis positioning, or recent cerebrovascular accidents were excluded from the study.

The temperature in the room where the intravenous fluid and irrigation fluids were stored was recorded on the days the fluids were needed for surgery. Each patient had his oral temperature monitored every 5 minutes during the procedure and immediately after prostatic resection, before he was taken out of the theatre. Their pulse rate, blood pressure and respiration were monitored. They were covered with theatre linen at room temperature during the procedure and were asked to indicate if they felt cold at any point during or after resection before their discharge. They were also observed for occurrence of shivering. If the patients in groups 1 and 2 complained of cold, they were externally warmed by covering them with warm blankets and commenced on warm intravenous infusion to achieve internal warming also. The duration of hospital stay after their surgery was also documented.

### Statistical analysis

The data was analyzed using the SPSS 11.0 for Windows. Comparison of means was carried-out using the Levene's statistical test and p-value ≤ 0.05 was considered as significant.

## Results

One hundred and twenty patients were studied. Table 1 shows the mean age, prostatic volume, resection time, weight of resected gland, room temperature, and the mean volume of irrigation fluid used in the 3 groups of patients. There was no significant difference between the 3 groups. The mean oral temperature at the commencement of the procedure was 36.6 ± 0.6 for each group. The mean decrease in oral temperature at the end of the procedure was greater in group 1 (0.98 ± 0.56°C) than in group 2 (0.42 ± .21°C). This difference was significant (p < 0.001). In group 3, there was a mean increase in temperature of 0.12°C but this was not significantly different from their immediate preoperative temperature (p > 0.05). Twenty two patients in group 1 felt cold compared to 5 patients in group 2 and none in group 3. This difference was significant (p < 0.001). Out of these patients who felt cold, shivering occurred significantly more in patients in group 1 (13 patients) than in patients in group 2 (3 patients) either towards the end or immediately after the procedure (p < 0.001). The mean post operative hospital stay was 8.3 ± 5, 6.1 ± 2 and 2 ± 0.7 hours for groups 1, 2, and 3 respectively. These were significantly different (p < 0.05)

**Table 1:** The mean age, prostatic volume, resection time, weight of resected gland, room temperature, and the mean volume of irrigation fluid used in the 3 groups of patients.

	Group 1 (n = 40)	Group 2 (n = 40)	Group 3 (n = 40)	P value
Mean age (yrs)	75.9 ± 20.2	76.1 ± 19.1	76 ± 21.2	> 0.05
Mean prostatic volume (cc)	59 ± 5	57 ± 8	58 ± 9	> 0.05
Mean resection time(mins)	51 ± 22	49 ± 17	55 ± 13	> 0.05
Mean wt of resected gland(gm)	11.7 ± 9	12.1 ± 7	12 ± 5	> 0.05
Mean room temperature°C	27 ± 5	27.3 ± 3	26.9 ± 5	> 0.05
Mean volume of irrigation fluid (L)	20.3 ± 7	22.2 ± 3	21.8 ± 1	> 0.05

## Discussion

The 3 groups of patients were matched for age, volume of the prostate gland, weight of the resected gland, volume of irrigation fluid used, resection time and the core body temperature at the commencement of the procedure and are therefore comparable (table 1).

The decrease in the mean core body temperature was significantly more in the patients who had resection with irrigation fluid and intravenous fluid at room temperature. More of them felt cold and subsequently shivered. These findings are in keeping with those from previous studies where significantly more patients developed perioperative hypothermia following the use of irrigation and intravenous fluids at room temperature during transurethral resection of their prostate gland [2,7,8,10,11]. Jaffe et al did not think that irrigation fluid temperature was a factor responsible for altering the core body temperature in their patients undergoing TURP, but the outcome of their study may have been affected by the fact that they actively warmed their patients externally during their study. Despite the use of isothermic irrigation in group 2 patients in this study, some drop in their core body temperature still occurred and some of them still felt cold and shivered though to a significantly less extent than in the patients who had their resection with irrigation and intravenous fluids at room temperature. This was also observed in previous studies [2,7,8,10,11,13]. However, when isothermic irrigation was combined with the use of warm intravenous fluids in group 3 patients, no significant alteration in the core body temperature was observed. None of the patients who received isothermic irrigation and warm intravenous fluid felt cold or shivered and their postoperative hospital stay was significantly shorter. We are aware that some previous studies utilized the Abbott level-one fluid warmer or the Ohio Servocare incubator to maintain fluid at the required temperature to the point of delivery into the body. None of these was available to us but we would like to believe that having them is unlikely to have materially altered the outcome of the study.

## Conclusion

We recommend that both the irrigation fluid and the intravenous fluids used during TURP should be isothermic. This will prevent the occurrence of perioperative hypothermia and may help reduce the occurrence of adverse cardiac events which contribute to the morbidity and mortality associated with TURP when irrigation fluid at room temperature is used.

## Competing interests

The author(s) declare that they have no competing interests.

## Pre-publication history

The pre-publication history for this paper can be accessed here:


